# ASO Author Reflections: Clinical Outcomes, Costs, and Value of Undergoing Surgery among Older Patients with Colon Cancer at *U.S. News & World Report* Ranked versus Unranked Hospitals

**DOI:** 10.1245/s10434-024-16274-w

**Published:** 2024-09-23

**Authors:** Abdullah Altaf, Timothy M. Pawlik

**Affiliations:** Department of Surgery, Division of Surgical Oncology, The Urban Meyer III and Shelley Meyer Chair for Cancer Research The, Ohio State University Wexner Medical Center and James Comprehensive Cancer Center, Columbus, OH USA

## Past

Previous studies have noted considerable variation in the safety of complex surgical procedures among different hospitals. Numerous quality indicators, including hospital volume, academic affiliation, and availability of specialized facilities, have been linked to variations in clinical outcomes among hospitals.^1^ As such, for patients with colon cancer, choosing the right hospital significantly increases the chances of receiving the best possible surgical care. A hospital’s reputation is one of the key factors that influences patient choice of treatment facility. National hospital rankings, such as the *U.S. News and World Report* (USNWR) rankings, enhance hospital reputations by emphasizing their dedication to safety and quality, thereby solidifying their “brand.” Widespread recognition of these rankings boost hospital visibility, increase patient volumes, and enhance financial outcomes for top-ranked hospitals.^3^ However, the association between USNWR hospital rankings and clinical outcomes, as well as the cost implications for complex procedures, remains ill-defined. To that end, the current study sought to quantify the varying effects of undergoing colon cancer surgery at USNWR-ranked versus unranked hospitals by comparing clinical outcomes, financial costs, and value of care.^2^

## Present

Among 184,327 Medicare beneficiaries who underwent surgery for colon cancer, 7.4% were treated at USNWR ranked hospitals, while 92.6% received care at 3573 unranked hospitals. All patients from the top 50 ranked hospitals (*n* = 13,650) were subsequently matched with an equal number of patients from 2522 (70.6%) unranked hospitals. Overall, ranked hospitals exhibited better clinical outcomes than unranked hospitals, with lower likelihood of 30-day mortality (2.13% versus 3.68%), failure-to-rescue (6.59% versus 10.48%), and 30-day readmission/death (15.46% versus 16.53%) (all *p* < 0.05). The improved outcomes were associated with a higher cost; specifically, mean adjusted cost of care at ranked hospitals was $55,996 versus $45,279 at unranked hospitals. Subsequent analyses stratified by risk quintiles (Q1 being the lowest-risk and Q5 being the highest-risk quintile) demonstrated that ranked hospitals outperformed unranked centers across various clinical outcomes as patient risk increased. The 30-day mortality difference widened from 0.53% in Q1 to 4.25% in Q5, indicating more of a survival benefit for higher-risk patients treated at ranked hospitals. Of note, as patient risk at admission increased, the cost disparity between ranked and unranked hospitals increased, with a concomitant higher reduction in 30-day mortality. In turn, this led to a roughly threefold decrease in the incremental cost-effectiveness ratio (ICER) from $9009 in the lowest-risk quintile to $3387 in the highest-risk quintile for a 1% reduction in mortality.

## Future

Treatment at USNWR-ranked hospitals, especially for higher-risk patients with colon cancer, yielded better clinical outcomes yet higher costs. The data underscore the need for targeted health policy reforms that prioritize equitable access to high-quality surgical care, which can be more expensive.^4^ As treatment at USNWR-ranked hospitals yielded better clinical outcomes, especially for higher-risk patients, additional measures are needed to bridge the quality gap between ranked and unranked hospitals.^4^ Policies should focus on standardizing care protocols across hospitals and enhancing resource allocation to unranked hospitals that serve high-risk populations. Moreover, transparency in hospital rankings and outcomes data should be used to empower patients in their hospital choice, as well as inform quality improvement projects with payers.^5^ Additionally, refining reimbursement models to reward hospitals for quality rather than quantity of care could incentivize unranked hospitals to improve their standards and align more closely with top-ranked institutions.^6^ Collectively, we need to strive for more just and equitable healthcare delivery systems in which hospital care quality is not linked to reputation but on high standards of care.
